# Hepatocyte IKK2 Protects Mdr2^−/−^ Mice from Chronic Liver Failure

**DOI:** 10.1371/journal.pone.0025942

**Published:** 2011-10-14

**Authors:** Hanno Ehlken, Vangelis Kondylis, Jan Heinrichsdorff, Laura Ochoa-Callejero, Tania Roskams, Manolis Pasparakis

**Affiliations:** 1 Institute for Genetics, Centre for Molecular Medicine (CMMC), Cologne Excellence Cluster on Cellular Stress Responses in Aging-Associated Diseases (CECAD), University of Cologne, Cologne, Germany; 2 Department of Pathology, Catholic University of Leuven, Leuven, Belgium; Université Paris Sud, France

## Abstract

Mice lacking the Abc4 protein encoded by the multidrug resistance-2 gene (*Mdr2* ^−/−^) develop chronic periductular inflammation and cholestatic liver disease resulting in the development of hepatocellular carcinoma (HCC). Inhibition of NF-κB by expression of an IκBα super-repressor (IκBαSR) transgene in hepatocytes was shown to prevent HCC development in *Mdr2* ^−/−^ mice, suggesting that NF-κB acts as a tumour promoter in this model of inflammation-associated carcinogenesis. On the other hand, inhibition of NF-κB by hepatocyte specific ablation of IKK2 resulted in increased liver tumour development induced by the chemical carcinogen DEN. To address the role of IKK2-mediated NF-κB activation in hepatocytes in the pathogenesis of liver disease and HCC in *Mdr2* ^−/−^ mice, we generated Mdr2-deficient animals lacking IKK2 specifically in hepatocytes using the Cre-loxP system. Mdr2^−/−^ mice lacking IKK2 in hepatocytes developed spontaneously a severe liver disease characterized by cholestasis, major hyperbilirubinemia and severe to end-stage fibrosis, which caused muscle wasting, loss of body weight, lethargy and early spontaneous death. Cell culture experiments showed that primary hepatocytes lacking IKK2 were more sensitive to bile acid induced death, suggesting that hepatocyte-specific IKK2 deficiency sensitized hepatocytes to the toxicity of bile acids under conditions of cholestasis resulting in greatly exacerbated liver damage. Mdr2^−/−^IKK2^Hep-KO^ mice remarkably recapitulate chronic liver failure in humans and might be of special importance for the study of the mechanisms contributing to the pathogenesis of end-stage chronic liver disease or its implications on other organs. ***Conclusion:*** IKK2-mediated signaling in hepatocytes protects the liver from damage under conditions of chronic inflammatory cholestasis and prevents the development of severe fibrosis and liver failure.

## Introduction

In humans, liver fibrosis is caused by a variety of different diseases such as chronic viral hepatitis, autoimmune liver disease and other disorders characterized by chronic liver inflammation [1]. Although the quality of the fibrosis may be different depending on the underlying pathology, in all cases fibrosis is caused by the activation of specialized stellate cells, which differentiate into active myofibroblasts that remodel the extracellular matrix of the liver [2], [3]. Under normal conditions myofibroblasts reside in a quiescent state and need to become stimulated in order to proliferate and differentiate into motile collagen-producing cells. In humans, almost all patients with chronic liver injury will develop fibrosis, which in many individuals will ultimately result in liver cirrhosis and end-stage liver disease, a condition that has a high morbidity and mortality and is discussed to favor initiation and progression of hepatocellular carcinoma [4]. Therefore, there is an urgent need to study the molecular and cellular mechanisms by which inflammatory signals regulate myofibroblast activation in order to better understand the mechanisms that control the pathogenesis of liver fibrosis and its detrimental consequences.

The multidrug resistance-2 knockout (Mdr2^−/−^) mouse provides a model for the study of liver fibrosis and hepatocarcinogenesis in the context of chronic inflammation. In contrast to other multidrug resistance proteins of the Mdr family, Mdr2 does not facilitate resistance towards specific chemicals (as for human Mdr1) by directly shuttling metabolites out of the cell [5]. Instead, studies in Mdr2^−/−^ mice suggest that Mdr2 mediates the flipping of phospholipids (phosphatidylcholine) from the cytosol of the hepatocyte into the bile canaliculi [5]. Phospholipids are thought to potently emulsify hydrophobic molecules such as certain bile acids and thereby attenuate their toxicity. Although the mechanisms controlling the pathogenesis of liver disease in Mdr2^−/−^ mice are still under discussion, several studies have demonstrated that mice lacking Mdr2 develop a chronic inflammatory liver condition, which is characterized by periductular inflammatory cell infiltration and periportal fibrosis typically bridging adjacent portal fields [6], [7], [8].^.^ Additionally, in the FVB/N genetic background, 100% of Mdr2 deficient mice develop hepatocellular carcinoma at the age of 16 months [8].

The transcription factor Nuclear Factor kappa B (NF-κB) regulates immune and inflammatory responses by controlling the expression of genes with important immunoregulatory functions. In resting cells NF-κB dimers are sequestered in the cytoplasm by association with inhibitory proteins belonging to the IκB family. When the cell is activated by a variety of stress-inducing stimuli, the IκB kinase complex (IKK), consisting of two catalytic subunits, IKK1 (or IKKα) and IKK2 (or IKKβ) and a regulatory subunit named NEMO (or IKKγ), phosphorylates IκBs on specific serine residues triggering their polyubiquitination and degradation through the proteasome. Consequently, NF-κB homo- and heterodimers are set free and translocate into the nucleus where they transactivate target genes, among them survival factors and inflammatory mediators [9], [10]. NF-κB is activated by a large number of stress-inducing stimuli, including cytokines, microbial products and conditions that impose threat on the cell such as radiation, hypoxia, mechanical stress [9]. With particular relevance to bile duct disease, cytotoxic bile acids are also potent activators of the NF-κB pathway [11], [12].

Studies in genetic mouse models revealed important functions of the IKK/NF-κB pathway in the regulation of liver physiology and the pathogenesis of liver diseases [9]. Mice lacking NEMO in liver parenchymal cells spontaneously develop chronic liver disease due to increased death of NEMO-deficient hepatocytes, which triggers liver inflammation and compensatory hepatocyte proliferation resulting in hepatocellular carcinoma (HCC) [13]. In addition, IKK2 has been shown to protect mice from chemically induced liver cancer mainly by inhibiting carcinogen-induced hepatocyte death and preventing compensatory proliferation of hepatocytes [14]. Moreover, recent studies showed that mice lacking both IKK1 and IKK2 in liver parenchymal cells develop severe jaundice and fatal cholangitis, demonstrating that IKK1 and IKK2 cooperate to maintain the integrity of the small bile ducts in the liver. Importantly, ablation of solely NEMO, IKK1 or IKK2 could not provoke this phenotype [15], indicating that the individual IKK subunits show a degree of functional redundancy in the liver.

NF-κB inhibition in hepatocytes by expression of an IκB super-repressor (IκBαSR) transgene did not affect liver inflammation in Mdr2^−/−^ mice but strongly reduced the development of hepatocellular carcinoma, suggesting that NF-κB activation promotes the survival of premalignant cells facilitating liver cancer development in this model [7]. To unveil the role of IKK2 in liver cancer development in Mdr2^−/−^ mice, we generated and analyzed Mdr2^−/−^ animals that lack IKK2 expression specifically in hepatocytes (Mdr2^−/−^IKK2^Hep-KO^). Surprisingly, we found that hepatocyte-specific deletion of IKK2 severely aggravated liver pathology of Mdr2^−/−^ mice. Mdr2^−/−^IKK2^Hep-KO^ mice developed severe jaundice at young age and failed to thrive remaining considerably smaller compared to their wild-type or *Mdr2* ^−/−^ littermates. In addition, Mdr2^−/−^IKK2^Hep-KO^ mice developed severe liver fibrosis accompanied by massive bile duct proliferation as compared to Mdr2^−/−^ mice. Finally, we show that IKK2 deficient hepatocytes were more sensitive to bile acid induced cytotoxicity, suggesting that IKK2 signaling has an important function to protect hepatocytes from bile acid toxicity and prevent severe liver damage in conditions of primary biliary disease.

## Results

### Mdr2^−/−^IKK2^Hep-KO^ mice develop severe wasting due to liver disease

**Figure 1:**
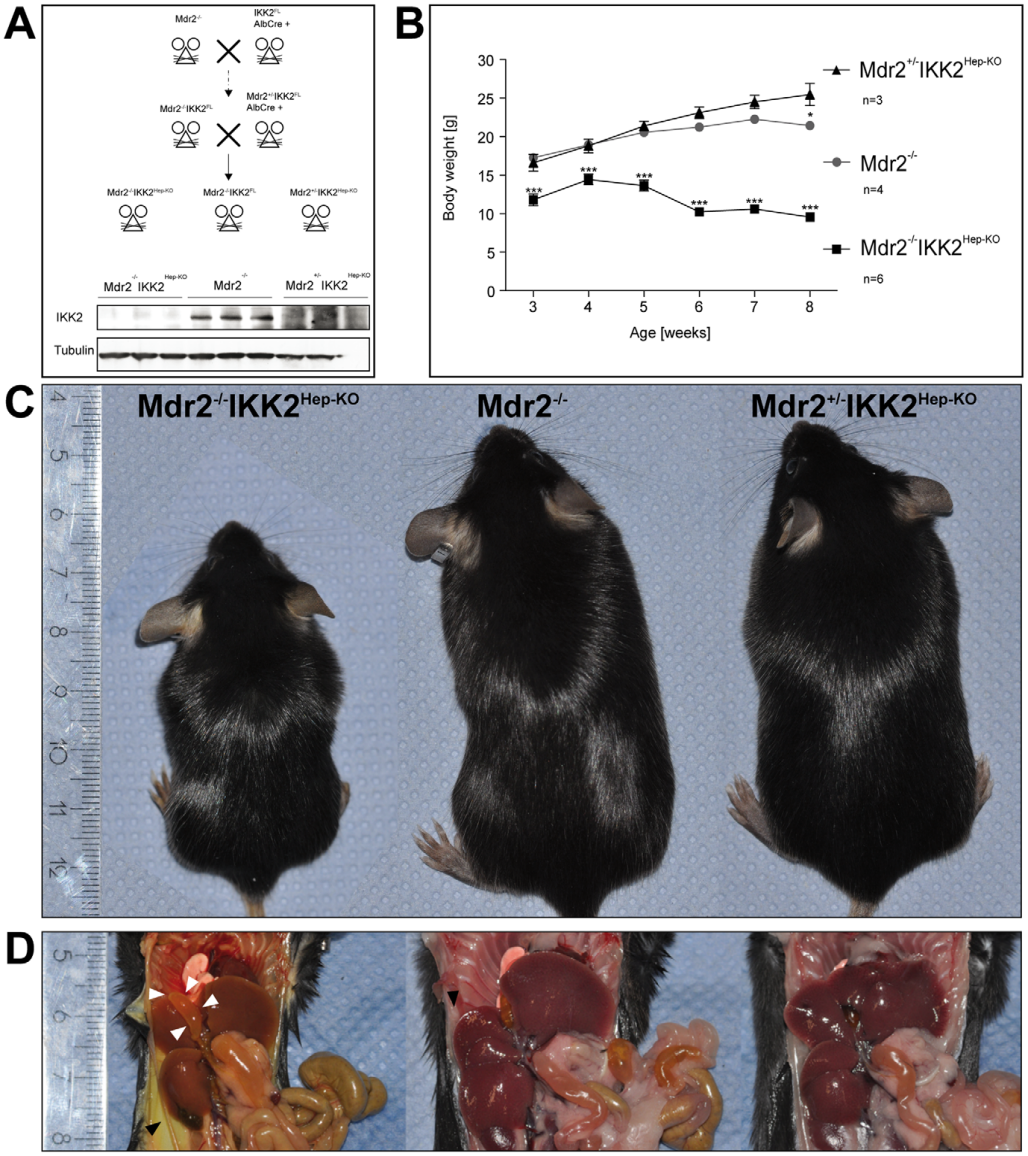
Mdr2^−/−^IKK2^Hep-KO^ mice show impaired growth and signs of jaundice. (A) Immunoblot of whole liver lysates from 3 representative mice for each depicted genotype. Tubulin serves as loading control. (B) Weight curve of male mice for the denoted genotypes. * p<0,05 for Mdr2^+/−^ IKK2^Hep-KO^ vs. Mdr2^−/−^, ** p<0,001 for Mdr2^−/−^ IKK2^Hep-KO^ vs. Mdr2^−/−^. (C) Macroscopic appearance of littermate male mice at 12 weeks of age. Genotypes as specified in the picture. (D) Necropsy of littermate mice from (C). White arrowheads: enlarged gall bladder in double knockout mouse. Black arrowhead: jaundiced peritoneal serosa in double knockout mouse vs. normal appearance of serosa in Mdr2^−/−^ and Mdr2^+/−^IKK2^Hep-KO^ mice. The ruler indicates centimeters in C and D.

To study the hepatocyte-specific role of IKK2 mediated NF-κB activation in liver disease and cancer induced by Mdr2 deficiency, we crossed Mdr2^−/−^ mice with mice carrying loxP-flanked IKK2 alleles [16] and the Albumin-Cre transgene [17]. These double Mdr2^−/−^IKK2^Hep-KO^ mice lack Mdr2 expression in all cells and additionally have a hepatocyte-specific ablation of IKK2 (Fig. 1A). Mdr2^−/−^IKK2^Hep-KO^ mice were born at the expected Mendelian ratio but were already distinguishable from their littermates in the first two weeks of life as they were considerably smaller. Although Mdr2^−/−^IKK2^Hep-KO^ mice showed an initial phase of growth till they reached 4 weeks of age, they subsequently lost about 30% of their body weight and at the age of 8 weeks weighed 50% less compared to their heterozygous Mdr2^+/−^IKK2^Hep-KO^ but also single Mdr2^−/−^ littermates (Fig. 1B, 1C). These results suggested that Mdr2^−/−^IKK2^Hep-KO^ mice suffer from liver disease resulting in poor growth and prompted us to investigate in detail the liver function in these animals.

Mdr2^−/−^IKK2^Hep-KO^ mice appeared severely jaundiced and their limbs and trunk showed varying degrees of muscle wasting and loss of fat mass (Fig. 1C). Necropsy of Mdr2^−/−^, Mdr2^+/−^IKK2^Hep-KO^ and Mdr2^−/−^IKK2^Hep-KO^ mice confirmed major jaundice of organs and serosae and substantial loss of body fat (Fig. 1D) only in the double knockout animals. The livers of Mdr2^−/−^IKK2^Hep-KO^ mice appeared yellow - green tainted and were harder to cut compared to the livers of Mdr2^−/−^ or Mdr2^+/−^IKK2^Hep-KO^ mice (Fig. 1D). Mdr2^−/−^IKK2^Hep-KO^ mice also showed massively enlarged gallbladders that were filled with bile (Fig. 1D). In contrast, single Mdr2^−/−^ and heterozygous Mdr2^+/−^IKK2^Hep-KO^ mice were indistinguishable from wild type mice (Fig. 1C, 1D) arguing that the lack of IKK2 in hepatocytes only culminates in the observed phenotype when Mdr2 is completely absent.

### Mdr2^−/−^IKK2^Hep-KO^ mice show increased liver damage

**Figure 2:**
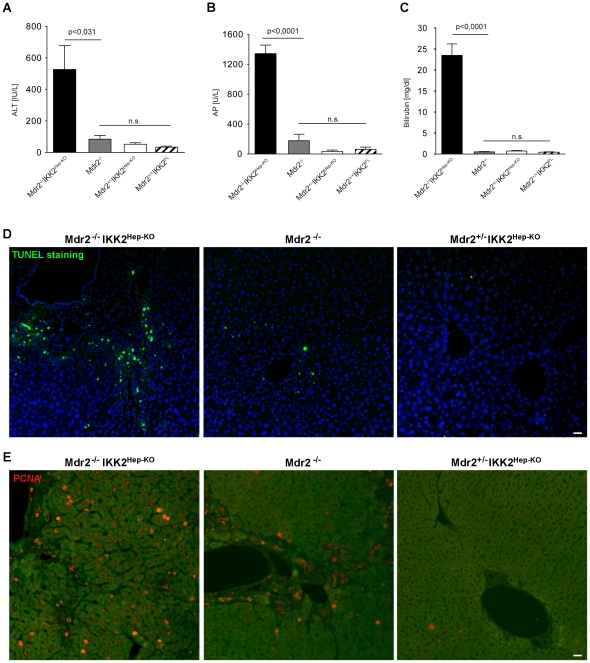
Mdr2^−/−^IKK2^Hep-KO^ mice exhibit increased liver damage, hepatocyte death and cholestasis. (A) Alanine aminotransferase (ALT), (B) alkaline phosphatase (AP), and (C) bilirubin levels in serum of adult male mice (8–26 weeks) with the indicated genotypes. Error bars indicate SEM. Number of mice: Mdr2^−/−^IKK2^Hep−^KO: n = 10, Mdr2^−/−^: n = 7, Mdr2^+/−^IKK2^Hep-KO^: n = 7 and Mdr2^+/+^IKK2^FL^: n = 2. (D–E) Representative pictures of TUNEL (D) and PCNA staining (E) on sections of paraffin-embedded livers from mice with the indicated genotypes. The green signal in E corresponds to background auto-fluorescence and was used to visualize the general morphology of the liver tissues. Scale bars: 20 µm.

To further characterize the liver disease developing in Mdr2^−/−^IKK2^Hep-KO^ mice we measured the levels of serum alanine amino transferase (ALT) in young adult mice (aged 2–6 months). We could not analyze a statistically meaningful number of mice in older age as most Mdr2^−/−^IKK2^Hep-KO^ mice died or showed very severe disease and had to be sacrificed before the age of 30 weeks. We found that Mdr2^−/−^IKK2^Hep-KO^ mice had about six-fold elevated serum ALT levels compared to their control littermates, indicating that these animals showed increased spontaneous liver damage (Fig. 2A). At this age, Mdr2^−/−^ mice did not show elevated serum ALT levels compared to heterozygous Mdr2^+/−^IKK2^Hep-KO^ mice. In line with the increased ALT levels, we found that liver sections from Mdr2^−/−^IKK2^Hep-KO^ mice contained increased numbers of dying hepatocytes as compared to Mdr2^−/−^ mice or Mdr2^+/−^IKK2^Hep-KO^ mice (Fig. 2D). In addition, liver sections from Mdr2^−/−^IKK2^Hep-KO^ also showed more PCNA positive cells in comparison to Mdr2^−/−^ mice or Mdr2^+/−^IKK2^Hep-KO^ mice indicating a higher level of compensatory proliferation (Fig. 2E). The observation that Mdr2^−/−^IKK2^Hep-KO^ mice were severely jaundiced prompted us to further investigate potential bile duct related complications. We therefore measured the levels of serum Alkaline Phosphatase (AP), a widely used marker for bile flow obstruction [18], in Mdr2^−/−^IKK2^Hep-KO^ and their control littermates. Indeed, serum AP levels were massively elevated in Mdr2^−/−^IKK2^Hep-KO^ mice compared to either Mdr2^−/−^ or Mdr2^+/−^IKK2^Hep-KO^ littermates (Fig. 2B). Additionally, Mdr2^−/−^IKK2^Hep-KO^ mice showed strongly elevated levels of total bilirubin in the serum compared to their littermate controls (Fig. 2C). These results suggest that Mdr2^−/−^IKK2^Hep-KO^ mice suffer from obstructive biliary disease. Mdr2^−/−^ mice showed slightly elevated AP levels compared to heterozygous Mdr2^+/−^IKK2^Hep-KO^ mice but the levels of bilirubin were within the normal range in both control groups. Collectively, these results showed that Mdr2^−/−^IKK2^Hep-KO^ mice spontaneously developed an obstructive bile duct-related disease resulting in liver damage and severe jaundice.

### Increased cholangiocyte proliferation and severe liver fibrosis in Mdr2^−/−^IKK2^Hep-KO^ mice

**Figure 3:**
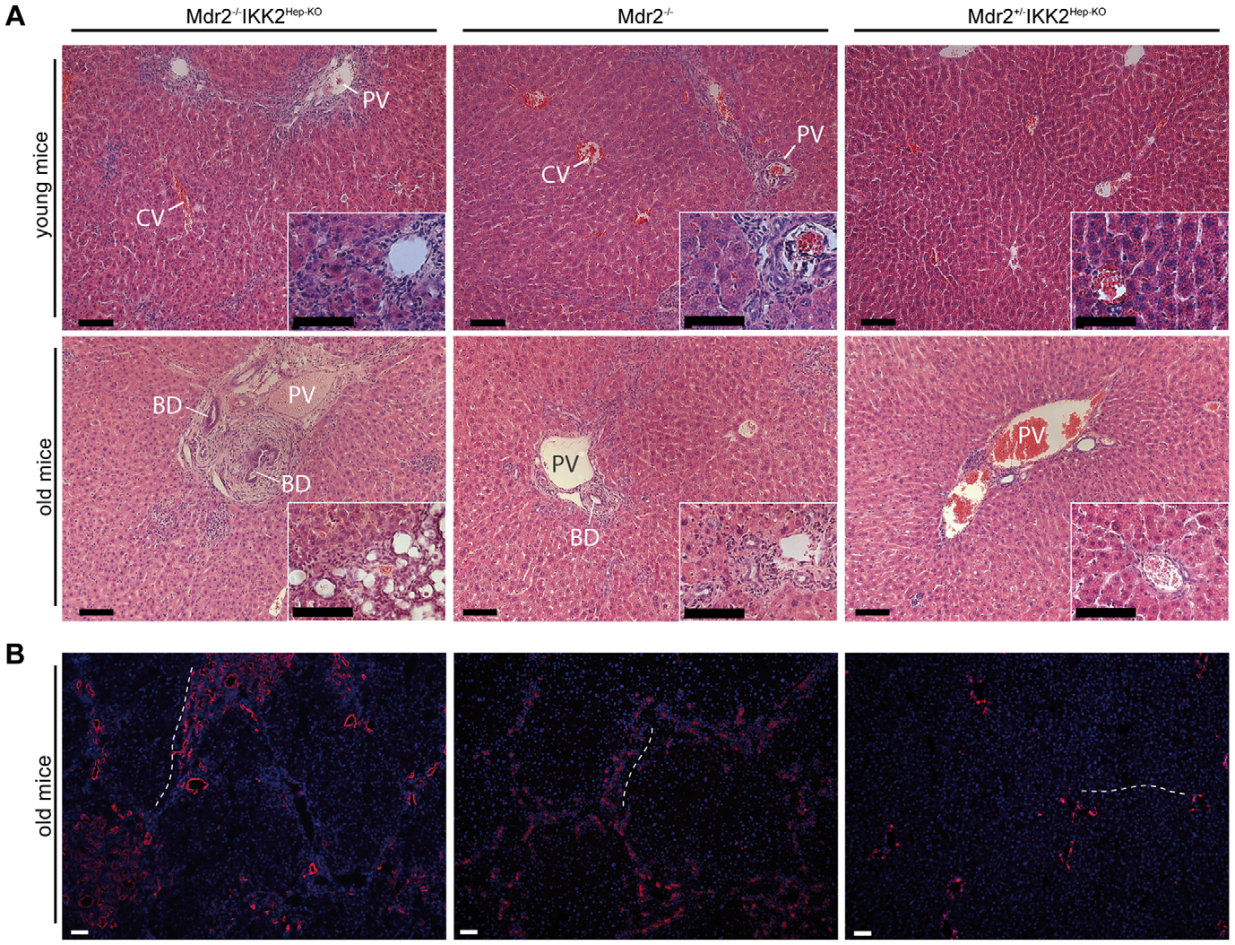
Histological analysis of the liver pathology in Mdr2^−/−^IKK2^Hep-KO^ mice. (A) Representative images of Hematoxylin & Eosin (HE) stained livers from young (12-week-old) littermate male mice and old (27–30 weeks) mice with the specified genotypes. Insert: High magnification of corresponding liver sections. (B) Immunofluorescence staining of cytokeratin 19 (CK19) in old male mice. DNA was stained with DAPI. The dashed line marks the distance from one portal field to the next, showing extreme abundance of CK19 positive cells in the Mdr2^−/−^ IKK2^Hep-KO^ as compared to Mdr2^−/−^ or Mdr2^+/−^IKK2^Hep-KO^ mice. PV portal vein. CV central vein. BD bile duct. Scale bars, 100 µm.

**Figure 4:**
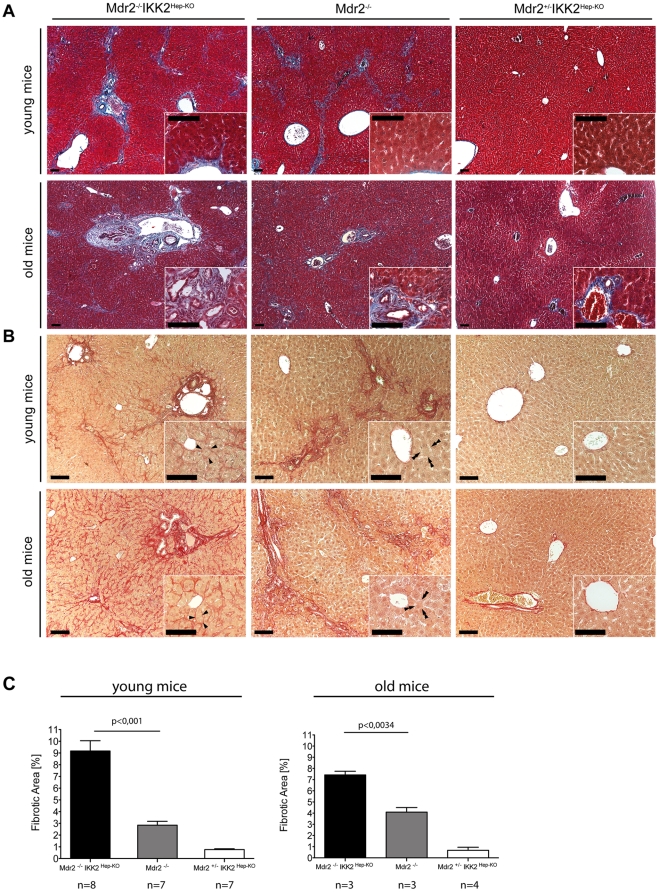
Mdr2^−/−^IKK2^Hep-KO^ mice develop severe fibrosis. (A) Representative images of Masson trichrome stained livers from 12-week-old littermate male mice and from 27–30 week-old male mice. (B) Representative images of Sirius Red stained livers from 12-week-old littermate male mice and from old adult male mice (27–30 weeks). Insert: High magnification of corresponding liver sections. Arrowhead: Pericellular fibers. Double arrowhead: Pericellular space free of stained fibers. Genotypes as in (A). (C) Quantification of fibrosis in mice with the depicted genotypes measured as percentage of Sirius Red positive area as a fraction of the total area of at least 10 high power fields per mouse. Left: young adult mice (males, 8–19 weeks), right: old adult mice (males, 20–42 weeks). Error bars indicate SEM. Scale bars, 100 µm.

To further characterize the liver pathology developing in Mdr2^−/−^IKK2^Hep-KO^ mice we performed immunohistochemical analysis of liver sections from these animals and from littermate controls. Consistent with previous reports [6], [8], [7], the livers of Mdr2^−/−^ mice showed immune cell infiltration around the bile ducts already at young age, which was more pronounced in older animals (Fig. 3A). Analysis of liver sections from Mdr2^−/−^IKK2^Hep-KO^ mice showed a more severe pathology with strong immune cell infiltration in the portal areas and massive expansion of the bile ducts in older animals (Fig. 3A). Immunostaining for cytokeratin 19, a marker of bile duct epithelial cells in the liver, revealed that Mdr2^−/−^IKK2^Hep-KO^ mice showed more pronounced expansion and hyperproliferation of biliary epithelial cells compared to Mdr2 deficient mice (Fig. 3B). Whereas the livers from Mdr2^−/−^ mice presented with hyperproliferating bile ducts close to or connecting the portal fields (Fig. 3A, 3B), Mdr2^−/−^IKK2^Hep-KO^ mice had strongly enlarged bile duct lumina and showed hyperproliferating bile ducts spanning from one portal field to the next also affecting more central areas (Fig. 3B and 4A). We did not detect histological signs of pathology in the livers of heterozygous *Mdr2* ^+/−^IKK2^Hep-KO^ control mice (Fig. 3A and 3B).

The livers from Mdr2^−/−^IKK2^Hep-KO^ mice were hard to cut, indicating the presence of fibrosis. We therefore stained liver sections with Masson's trichrome and with Sirius red to visualize fibrotic areas. As reported previously, livers from Mdr2^−/−^ mice showed bridging fibrosis with connective tissue fibers concentrating in the periportal and periductular area as shown by Masson Trichrome and Sirius Red staining (Fig. 4A and 4B respectively). Interestingly, fibers were more abundant in the liver of Mdr2^−/−^IKK2^Hep-KO^ mice already at younger age compared to Mdr2 knockouts, while Mdr2^+/−^IKK2^Hep-KO^ animals did not show any fibrosis (Fig. 4A and 4B). Furthermore, already at young age fibrosis in Mdr2^−/−^IKK2^Hep-KO^ mice followed also a pericellular distribution (Fig. 4B). This latter pattern of fiber deposition is sometimes referred to as chicken-wire fibrosis and in humans can help to distinguish between causes of liver injury [19]. Only in Mdr2^−/−^IKK2^Hep-KO^ animals fibrosis reached a stage where macroscopically and microscopically the liver surface became uneven (data not shown). Quantification of the fibrotic area in Sirius red stained liver sections revealed that Mdr2^−/−^IKK2^Hep-KO^ mice have significantly more fibrosis as compared to Mdr2^−/−^ and Mdr2^−/−^IKK2^Hep-KO^ mice (Fig. 4C).

Mdr2^−/−^ mice in the FVB/N genetic background were shown to spontaneously develop hepatocellular carcinoma with 100% penetrance by the age of 16 months [8]. Due to the severe phenotype and early lethality of Mdr2^−/−^IKK2^Hep-KO^ mice we could not analyze animals older than 9–10 months to evaluate HCC development. However, we did not observe any malignant or premalignant lesions in livers from Mdr2^−/−^IKK2^Hep-KO^ at 2 to 10 months of age. In addition, we could not detect any malignant or premalignant areas in the livers of Mdr2^−/−^ mice up to 14 months of age (data not shown). These findings are consistent with a recent report demonstrating that when Mdr2^−/−^ mice were crossed into the C57Bl/6 background tumor development was suppressed [20].

### IKK2 protects primary hepatocytes from bile acid induced apoptosis

Our results presented above showed that hepatocyte-specific ablation of IKK2 severely affected liver function in Mdr2-deficient animals resulting in chronic obstructive bile duct disease, severe jaundice, liver fibrosis and early death.

**Figure 5:**
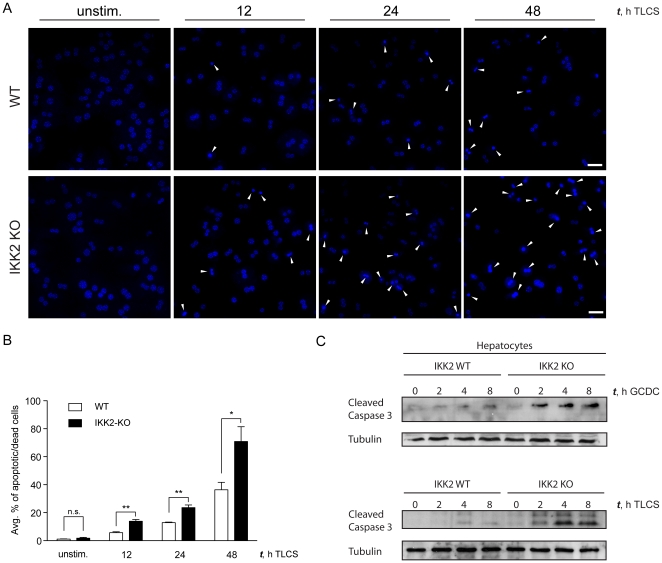
IKK2-deficient hepatocytes are more sensitive to bile acid induced apoptosis compared to wild type cells. (A) Representative images of DAPI stained primary WT and IKK2-KO hepatocyte cultures. Cells were left untreated or were stimulated with taurolithocholic acid 3-sulfate disodium salt (TLCS, 100 µM) for the indicated time periods. Arrowheads show apoptotic hepatocytes as indicated by smaller highly fluorescent nuclei with condensed chromatin. Scale bars, 100 µm. (B) Graphs showing the average proportion of apoptotic hepatocytes from 3 independent experiments. Mean values ± SEM shown. * p<0,05; ** p<0,01; n.s.: not statistically significant. (C) Immunoblot detection of cleaved Caspase-3. Primary hepatocytes were stimulated with glycochenodeoxy-cholate (GCDC, 50 µM, top) or TLCS (100 µM, bottom) for the indicated time periods. Tubulin serves as loading control.

Mdr2-deficiency impairs the enrichment of bile with phospholipids, which are important to emulsify and thus reduce the toxicity of bile acids. Therefore the bile of Mdr2^−/−^ mice is more toxic. We hypothesized that IKK2 ablation in hepatocytes strongly aggravated the liver pathology of Mdr2-deficient mice by sensitizing hepatocytes to bile acid toxicity. Indeed, bile acids have been shown to activate the NF-κB pathway in obstructive cholestasis suggesting that NF-κB might have a protective function in cholestatic liver disease, although the exact mechanism remains poorly understood [12]. It has been shown that hydrophobic bile acids can induce apoptosis in primary cultured hepatocytes of mouse or rat origin [21]. We therefore chose to compare the toxicity of glycochenodeoxycholate (GCDC) and taurolithocholylsulfate (TLCS), both of which have been reported to induce apoptosis in primary hepatocytes from rat and mouse [11], [12], [22], [23], [24], in wild-type and IKK2-deficient primary hepatocytes. TLCS treatment of wild-type and IKK2-deficient primary hepatocytes resulted in markedly increased numbers of apoptotic/dead cells as revealed after DAPI staining by the detection of small highly fluorescent nuclei with condensed chromatin [25] (Fig. 5A). We observed about two-fold increase of apoptotic/dead cells in the IKK2-deficient as compared to the wild-type hepatocytes at 12, 24 and 48 hours of stimulation with TLCS (Fig. 5B). To assess differences in the early apoptotic response we stimulated wild type and IKK2-deficient primary mouse hepatocytes with TLCS or GCDC and examined caspase-3 activation by immunoblotting with antibodies recognizing the cleaved/active version of caspase-3. These experiments revealed a stronger and earlier activation of caspase-3 in TLCS and GCDC stimulated IKK2-deficient primary hepatocytes, suggesting that NF-κB inhibition by ablation of IKK2 sensitized hepatocytes to bile acid-induced apoptosis (Fig. 5C). Taken together, these results show that IKK2-mediated signaling protects primary hepatocytes from the toxicity of hydrophobic bile acids, suggesting that NF-κB activation in hepatocytes is critical for the prevention of liver damage in obstructive biliary disease.

## Discussion

In humans, chronic cholestatic liver diseases constitute a major risk for the development of liver cirrhosis and end-stage liver disease culminating in liver failure. Obstruction of the bile flow in bile ducts and bile canaliculi can damage both bile duct epithelial cells and hepatocytes. Our knowledge about the mechanisms regulating the cross-talk between hepatocytes and bile duct epithelial cells in the normal liver and also under conditions of cholestasis remains limited. The Mdr2 knockout mouse is an excellent model for the study of chronic inflammatory biliary liver disease, liver fibrosis and hepatocarcinogenesis. Abc4, the product of the Mdr2 gene, is expressed in hepatocytes and functions to flip phospholipids into the bile canaliculi. Phospholipids emulsify hydrophobic bile acids reducing their toxic effects, therefore enrichment of the bile with phospholipids is important to reduce bile toxicity. The increased toxicity of the bile in Mdr2-deficient mice is thought to induce bile duct inflammation and the liver disease in these animals [6], [5]. Here we show that hepatocyte-specific ablation of IKK2 in the context of disturbed bile homeostasis induced by Mdr2 deficiency results in severe cholestatic liver disease in young age, suggesting that activation of the IKK2/NF-κB pathway in hepatocytes constitutes an essential regulator of liver function under conditions of bile duct inflammation.

In older Mdr2^−/−^ mice, bile duct inflammation progresses to fibrosis especially around the periportal area of the liver lobule. Ultimately, as a result of the chronic inflammation, Mdr2^−/−^ mice in the FVB genetic background develop hepatocellular carcinoma by the age of 16 months [26]. Mdr2^−/−^IKK2^Hep-KO^ mice showed more severe bile duct inflammation and biliary epithelial cell hyperproliferation, and also they presented with more severe fibrosis in all areas of the liver, i.e. periportal and pericentral areas compared to Mdr2^−/−^ animals. At early stages, Mdr2^−/−^IKK2^Hep-KO^ mice also showed more prominent fibrosis in the periportal area as compared to the central area of the liver lobule. It seems plausible that while in Mdr2^−/−^ mice hepatocyte damage is restrained in the vicinity of the bile ducts, the absence of IKK2 in Mdr2^−/−^IKK2^Hep-KO^ hepatocytes facilitates the extension of parenchymal damage spreading into more centrally located areas of the liver lobule. Our experiments showing that IKK2-deficient primary hepatocytes were more sensitive to apoptosis induced by hydrophobic bile acids, suggest that low concentrations of toxic bile acids, which might not reach the threshold required to damage wild-type hepatocytes, could trigger the death of IKK2-deficient cells. This increased sensitivity of IKK2-deficient hepatocytes to bile acid toxicity could explain the spreading of the damage and the severe liver disease in the Mdr2^−/−^IKK2^Hep-KO^ mice.

The original aim of our experiments was to address the role of IKK2-mediated NF-κB activation in hepatocytes in HCC development in Mdr2^−/−^ mice, prompted by the study of Pikarsky et al. [7] who showed that NF-κB inhibition in hepatocytes by expression of an IκBα super-repressor could prevent or delay HCC development in Mdr2-deficient mice. In particular since IKK2^Hep-KO^ mice were shown to develop more liver tumors than wild type mice in response to administration of the chemical carcinogen diethylnitrosamine (DEN), we were curious to test whether IKK2 deficiency would ameliorate or aggravate HCC development in Mdr2^−/−^ mice. However, we found that hepatocyte-specific IKK2 ablation caused a severe cholestatic liver disease already in very young Mdr2^−/−^ mice resulting in poor growth and early lethality, which prevented the study of HCC development as the double knockout mice did not live long enough to analyze tumor development. The oldest Mdr2^−/−^IKK2^Hep-KO^ mouse we obtained was 42 week old and had no signs of HCC development. Moreover, histological evaluation failed to reveal even the earliest premalignant lesions that have been described to occur in the liver of Mdr2^−/−^ mice at younger age [6] in our Mdr2^−/−^IKK2^Hep-KO^ mice (data not shown). However, histological examination of Mdr2^−/−^ mice up to 14 months of age failed to reveal any evidence of malignant or premalignant lesions (data not shown). As the *Mdr2* ^−/−^ mice in our colony were backcrossed for at least 4 generations in the C57BL/6 genetic background, it is likely that the C57Bl/6 genetic background prevents HCC development in these mice in contrast to the FVB/N strain in which the malignant phenotype was described [6]. The absence of tumors in our *Mdr2* ^−/−^ mice (having more than 90% C57Bl/6 genetic background) is consistent with the results of Klopstock et al, who observed strong reduction in HCC development in *Mdr2* ^−/−^ mice having 75% FVB/N and 25% C57Bl/6 genetic background [20]. The C57Bl/6 genetic background does not prevent HCC development only in the *Mdr2* ^−/−^ but also in the HCV/ATX mouse model as first described by Keasler et al. [27].

Curiously, Pikarsky et al. showed that NF-κB inhibition in hepatocytes did not affect the early liver disease in Mdr2^−/−^ mice [7]. In contrast, our results presented here show that IKK2-ablation strongly aggravated the liver pathology of Mdr2^−/−^ mice already at a very young age. This apparent discrepancy could be due to the different genetic background of the animals, as our studies were performed in mice backcrossed into the C57Bl/6 genetic background while Pikarsky et al. studied animals in the FVB/N background. Alternatively, qualitative or quantitative differences in the NF-κB inhibition achieved by the two different models could also affect the phenotype. IKK2-ablation may have a stronger inhibitory effect as compared to the expression of the IκBα super-repressor transgene. Another possibility is that IKK2 knockout prevents the nuclear translocation of a broader range of NF-κB dimers compared to the IκBα-SR transgene, which potently inhibits p50/p65 heterodimers but may be less efficient to prevent nuclear translocation of other dimers. Finally, we cannot exclude the possibility that additional non-NF-κB related functions of IKK2 might also contribute to the observed phenotype in Mdr2^−/−^IKK2^Hep-KO^ mice. Further experiments will be required to resolve the differential effect of IKK2 ablation versus IκBα super-repressor expression in *Mdr2* ^−/−^ mice.

Interestingly, Mair et al. recently demonstrated that Mdr2^−/−^ mice with liver parenchymal cell specific deficiency of STAT3 developed an aggravated liver disease compared to Mdr2^−/−^ mice. Mdr2^−/−^ mice lacking STAT3 or STAT5 in hepatocytes strikingly resemble the phenotype of Mdr2^−/−^IKK2^Hep-KO^ mice [28], [29]. In one study the absence of STAT3 rendered primary hepatocytes more vulnerable to apoptosis induced by bile acids as we have observed in our experiments with IKK2 deficient hepatocytes [28]. Thus, NF-κB, STAT3 and STAT5 are important to protect hepatocytes from bile acid toxicity and prevent early liver damage in the Mdr2^−/−^ genetic background.

In conclusion, our results revealed a previously unrecognized essential function of IKK2-mediated signaling to protect hepatocytes from bile acid toxicity and prevent or ameliorate liver damage under conditions of inflammatory biliary disease. Mice lacking both Mdr2 and IKK2 in hepatocytes developed a severe liver disease characterized by cholestasis, major hyperbilirubinemia and severe to end-stage fibrosis, which resulted in muscle wasting, loss of body weight, lethargy and spontaneous death. Thus, Mdr2^−/−^IKK2^Hep-KO^ mice remarkably recapitulate chronic liver failure in humans and might be of special importance for the study of the mechanisms contributing to the pathogenesis of end-stage chronic liver disease or its implications on other organs.

## Materials and Methods

### Animal experimentation

All animal procedures were conducted in accordance with European (EU directive 86/609/EEC), national (TierSchG), and institutional guidelines, and protocols and were approved by local governmental authorities (Landesamt für Natur, Umwelt und Verbraucherschutz Nordrhein-Westfalen) under the license 8.87–50.10.37.09.242.

The general health status of the mice was monitored regularly and assessed by the following criteria: body weight, posture, signs of pain, distress or discomfort and insufficient grooming. In order to minimize suffering, mice were sacrificed when showing deterioration in their general health. C57Bl/6 mice carrying loxP-site flanked ikk2 alleles (IKK2^FL^) [16] were crossed to Albumin-Cre transgenic mice [17] to obtain mice with a hepatocyte specific deletion of IKK2 (IKK2^Hep-KO^). IKK2^Hep-KO^ mice were crossed to Mdr2^−/−^ mice to generate double knockout mice (Mdr2^−/−^IKK2^Hep-KO^), mice heterozygous for Mdr2 carrying liver-specific deletion of IKK2 (Mdr2^+/−^IKK2^Hep-KO^) and also *Mdr2* ^−/−^ mice carrying the IKK2 loxP-flanked alleles but not expressing Cre recombinase (Mdr2^−/−^IKK2^FL/FL^, referred to as Mdr2^−/−^ in the text and figures).

### Genotyping

Genotyping-PCR was performed on DNA prepared from tail biopsies of mice. The following primers were used for typing of IKK2^floxed^ and Mdr2^−/−^ and Mdr2^+/−^ mice: Mdr2: 5′- CGGCGAGGATCTCGTCGTGACCCA-3′, 5′-TGATGAATATTGGCGTTGTG-3′, 5′-AATAGGGCAAACAGTCTACAG-3′. IKK2: 5′ - GTT CAG AGG TTC AGT CCA TTA TC - 3′, 5′ - TAG CCT GCA AGA GAC AAT ACG - 3′, 5′ - TCC TCT CCT CGT CAT CCT TCG - 3′. CRE: 5′ – GTC CAA TTT ACT GAC CGT ACA C - 3′, 5′- CTG TCA CTT GGT CGT GGC AGC - 3′.

### Histochemistry and Immunofluorescent Liver stainings

Hematoxylin/Eosin (HE), Masson's trichrome and Sirius red staining of liver sections were performed as recommended by the manufacturer of the respective chemicals or staining kits (Sigma Aldrich). Immunofluorescent detection of Cytokeratin 19 (CK19) was performed on 10–12 µm thick freshly cut cryosections from liver pieces previously kept on −80°C in tissue freezing medium (Jung, Leica Microsystems Nussloch GmbH, Germany). After fixation for 10 min in 4°C acetone and washing in PBS, sections were incubated in blocking buffer (PBS, 3% BSA, 5% FCS) for 60 min. The sections were then incubated overnight at 4°C with Cytokeratin 19 antibody (goat, Santa Cruz, 1∶180 in blocking solution). After washing, the secondary antibody (Alexa Fluor 594 donkey anti goat, molecular probes) was applied (1∶1000 in PBS, 0.5% BSA) at room temperature for 60 minutes. After washing, sections were incubated with DAPI and mounted with fluoromount. All images were analyzed and acquired on a Leica DM 5500 B microscope system.

### TUNEL staining

Dying hepatocytes were detected by the terminal deoxynucleotidyl transferase-mediated nick-end labeling (TUNEL) staining kit according to the manufacturer's instructions (Promega).

### PCNA staining

Paraffin-embedded 5 µm sections were used for PCNA staining after antigen retrieval by heating in citrate buffer [13]. The mouse anti-PCNA antibody (Invitrogen) was used at a 1∶200 dilution followed by a goat anti-mouse secondary antibody coupled to Alexa 594 (Molecular probes) at a 1∶300 dilution. The samples were visualized under a Zeiss LSM510 confocal microscope.

### Biochemical analysis

Alanine aminotransferase, bilirubin and alkaline phosphatase were measured on a Cobas chemical analyzer unit according to the manufacturer (Roche Diagnostics, Mannheim, Germany). Whole blood was collected by direct puncture of the heart and the serum was obtained by 20-minute centrifugation at 13000 rpm.. The samples were diluted 1∶6 (NaCl 0,9%) for measurements. The results in graphs are depicted multiplied by 6.

### Quantification of fibrotic area

The fibrotic area was measured by quantification of Sirius red stained liver tissue relative to the section area. Therefore, at least 10 liver sections per mouse (at 100× magnification) from 2–3 liver lobes were cut at 5 µm and stained with Sirius red. The imageJ software was utilized to measure Sirius red positive tissue area.

### Isolation of primary hepatocytes and cell culture experiments

Anesthetized mice were perfused via the vena cava with solution I (EBSS without Ca^2+^ and Mg^2+^, 0,5 mM EGTA). Subsequently, perfusion with 50 ml of collagenase solution (EBSS with Ca^2+^ and Mg^2+^, 10 mM Hepes, 3810 U collagenase and 2 mg Trypsin inhibitor) was performed and single cell suspensions of perfused liver were generated using a 70 µm nylon mesh. Hepatocytes were washed twice in high glucose DMEM supplemented with 1% FCS. 2,5×10^6^ cells were seeded on collagen coated 10 cm dishes and after 4 hours the medium was renewed. Cells were stimulated the day after plating for 2, 4 and 8 hours with hydrophobic bile acids. Sodium glycochenodeoxy-cholate (Sigma G0759) was prepared as 1000× Stock in DMSO and added to the hepatocytes in DMEM/1% FCS to a final concentration of 50 µM. Taurolithocholic acid 3-sulfate disodium salt (Sigma T0512) was prepared as 1000× Stock in Methanol and added to the hepatocytes in DMEM/1% FCS to a final concentration of 100 µM. For dead cell counts after stimulation with bile acids primary hepatocytes were plated on fibronectin (BD Bioscience) covered Menzel glasses in 6 well plates and stimulated with taurolithocholic acid 3-sulfate disodium salt (TLCS, Sigma) for the indicated time periods. Apoptotic nuclei were identified by DAPI staining (as indicated by smaller highly fluorescent nuclei with condensed chromatin) [25]. Three independent experiments were performed and apoptotic/dead and total number of cells were counted in 3 random non-overlapping high power fields per timepoint. Unstimulated primary hepatocytes were changed to fresh DMEM/1% FCS medium and kept until the 48 h time point and then further processed as the stimulated cells.

### Immunoblot analysis

Cells were lysed with NP40 buffer supplemented with protease- and phosphatase inhibitors (both Roche, Mannheim Germany). 5× SDS sample buffer was added, extracts were boiled for 10 minutes and run on standard 10% or 15% SDS polyacrylamid gels. The following antibodies were used in this study: α-IKK2: cell signaling #2684, 1∶1000, α-cleaved caspase-3 (Asp175): cell signaling, #9664, 1∶1000, α-Tubulin: Sigma, #T6074, 1∶1000, Alkaline phosphatase conjugated anti-rabbit (NA934V) and anti-mouse (NA9310V) antibodies were both from GE Healthcare and were used 1∶10.000 and 1∶5000 respectively.
